# Highly Sensitive Colorimetric Detection of Ochratoxin A by a Label-Free Aptamer and Gold Nanoparticles

**DOI:** 10.3390/toxins7124883

**Published:** 2015-12-10

**Authors:** Yunxia Luan, Jiayi Chen, Cheng Li, Gang Xie, Hailong Fu, Zhihong Ma, Anxiang Lu

**Affiliations:** 1 Agriculture Environment, Beijing Research Center for Agricultural Standards and Testing, Beijing Academy of Agriculture and Forestry Sciences, Beijing 100097, China; luanyx@nercita.org.cn (Y.L.); cjy_1666@126.com (J.C.); lic@nercita.org.cn (C.L.); fhailong84@126.com (H.F.); mazh@nercita.org.cn (Z.M.); 2 Risk Assessment Lab for Agro-products (Beijing), Ministry of Agriculture, Beijing 100097, China; 3 Grain Safety, Academy of State Administration of Grain, Beijing 100037, China; wsx@chinagrain.org

**Keywords:** aptamer, Ochratoxin A (OTA), gold nanoparticles, cationic polymer, colorimetric assay

## Abstract

A label-free aptamer-based assay for the highly sensitive and specific detection of Ochratoxin A (OTA) was developed using a cationic polymer and gold nanoparticles (AuNPs). The OTA aptamer was used as a recognition element for the colorimetric detection of OTA based on the aggregation of AuNPs by the cationic polymer. By spectroscopic quantitative analysis, the colorimetric assay could detect OTA down to 0.009 ng/mL with high selectivity in the presence of other interfering toxins. This study offers a new alternative in visual detection methods that is rapid and sensitive for OTA detection.

## 1. Introduction

Ochratoxin A (OTA), a polyketide-derived secondary metabolite of *Aspergillus* and *Penicillium* strains, is a type of mycotoxin presents in grains, nuts, cottonseed and other commodities associated with agricultural products and animal feeds [1,2,3,4]. OTA is a small molecule which can cause immunosuppression and is weakly mutagenic as well as immunotoxic [5]. OTA is regarded as a potential carcinogen by the International Agency for Research on Cancer (IARC) [6]. As far as protection of consumers’ health is concerned, maximum residue limits (MRL) for OTA in foods and raw products have been established by the governments of many countries. The Codex Alimentarius Commission (CAC) has adopted an MRL of 5.0 μg/kg for OTA in food while the MRLs for different foods are in the range of 0.5–20.0 μg/kg in China [7,8].

For accurate and sensitive detection of OTA residues in food, thin layer chromatography (TLC) [9], high-performance liquid chromatography (HPLC) [10], gas chromatography (GC) [11], ultraviolet-visible, fluorescence and mass spectrometry (MS) [10,12], and enzyme-linked immunosorbent assay (ELISA) have been used [13]. Although these methods are the most commonly used, their high sensitivity and selectivity are coupled with the high costs of sophisticated equipment. Highly trained personnel are also required and the methods are not cost-effective, requiring a relatively long analysis time, so they are neither readily available in developing countries nor capable of on-site detection. Recently, some rapid detection methods based on immunoassays, such as ELISA, have been applied in mycotoxin residue detection. However, because of the difficulties in preparation of monoclonal antibodies and the limitations of proteins, these methods may be susceptible to the surrounding conditions. Therefore, it is still highly desirable to develop simpler and more sensitive methods to detect trace OTA in different samples.

Aptamers are single-stranded DNA or RNA that can recognize small molecules, proteins, and multiple metal ions [14,15,16,17,18]. Target-specific aptamers are engineered by the systematic evolution of ligands by exponential enrichment (SELEX) [19]. The technique of selecting aptamers was reported by Ellington and Gold in 1990 [20]. Aptamers not only show a high affinity and specificity for their target ligands but also exhibit excellent stability and wide applicability [21]. These properties make aptamers suitable for use in medical diagnosis, environmental monitoring and biological analysis [22,23]. Recently, there has been a tremendous increase in reports on aptamer-based biosensors (aptasensors) for OTA detection. A variety of analytical techniques based on aptamers have been developed, including colorimetric assay, fluorescence assay, and electrochemical aptasensor [24,25,26]. Among these methods, the analysis based upon colorimetric assay has the advantages of simplicity, rapidity, lower cost and more suitability for on-site detection. Besides, many assays require the aptamer to be labeled, which would not only make experiments relatively more expensive and complex, but may also affect the binding affinity between the OTA and aptamer and influence the sensitivity for detection [27,28,29]. Therefore, new analyses, especially those rapid, simple, sensitive and cost-effective methods, are highly desired for quantitative OTA detection.

Herein, we develop an aptamer-based label-free approach to detect OTA using the cationic polymer poly diallyldimethylammonium chloride (PDDA) in the polymer-mediated aggregation of gold nanoparticles (AuNPs) [30]. PDDA is a cost-effective polymer with high sensitivity, better than salt with high concentration and other polymers. OTA was detected by monitoring the chromatic change of the AuNPs with the naked eye. This method is simple, rapid, and highly sensitive and extends the available detection methods for OTA.

## 2. Experimental

### 2.1. Reagents and Apparatus

OTA aptamer (5′-CTGGGAGGGAGGGAGGGATCGGGTGTGGGTGGCGTAAAGGGAGCATCGGACACCCGATCCC-3′) oligonucleotide was synthesized and then purified by HPLC (Sangon Biotechnology Co. Ltd., Shanghai, China) according to Cruz-Aguado and Penner [31]. HAuCl_4_, sodium citrate and Tris-HCl were purchased from Sigma-Aldrich (St. Louis, MO, USA). Poly (diallyldimethylammonium chloride) (PDDA) was obtained from Sigma-Aldrich. All reagents were of analytical grade and the solutions were prepared with Tris-HCl buffer solution (pH 7.4). Ultraviolet-visible (UV-vis) absorbance spectra were recorded by a TU-1901/TU-1900 UV-vis spectrometer (Purkinje General, Beijing, China). Ultrapure water (Milli-Q plus, Millipore Inc., Billerica, MA, USA) was used throughout all experiments.

### 2.2. Preparation of AuNPs

All glassware were soaked in 1:3 (*v*/*v*) HNO_3_–HCl, followed by rinsing with ultrapure water and drying in an oven. AuNPs solutions were then synthesized by sodium citrate reduction of HAuCl_4_ [32]. In brief, 2 mL of sodium citrate was added to a boiling solution of 1 mM HAuCl_4_ with magnetic stirring [33]. The solution was heated for a further 20 min after changing color from grey to wine red. The solution was stirred until the temperature had dropped to room temperature. The resulting AuNPs solutions were stored in dark bottles at 4 °C.

### 2.3. Colorimetric Detection of Ochratoxin A

First, 500 μL of 5 nM PDDA (dissolved in OTA binding buffer consisting of 50 mM Tris-HCl, 120 mM NaCl, 5 mM KCl and 20 mM CaCl_2_) was mixed with 1 μL of 50 μM OTA aptamer in a 1.5 mL plastic tube. After incubation for 5 min, 500 μL of AuNPs solution was added. After a further 5 min, an appropriate volume and concentration of OTA was added into the solution and incubated for 20 min. Finally, the resulting solution was transferred into a 1 cm micro-quartz cuvette for spectral recording. The developed label-free aptamer-based assay was used for the determination of OTA in Mao-tai liquor, a famous distilled Chinese liquor made from wheat and sorghum. Aliquots were 100-fold diluted with OTA binding buffer. Different amounts of OTA standard solution in methanol were added into 1% liquor to obtain diluted liquor samples contaminated with OTA at 0.05, 0.1, 0.5, 1, 5, 10, 50 ng/mL. The absorbance value was recorded at 520 nm.

## 3. Results and Discussion

### 3.1. Principles of the Colorimetric Method for Ochratoxin A Detection

**Scheme 1:**
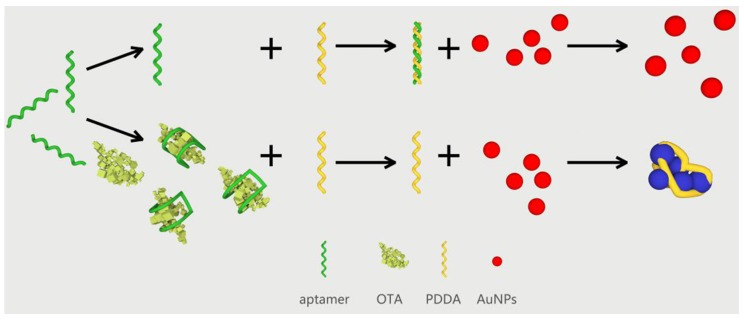
Mechanism for the poly diallyldimethylammonium chloride (PDDA)-induced aggregation of AuNPs in Ochratoxin A (OTA) detection.

PDDA is a water-soluble cationic polymer, and serves a dual function including aggregation of AuNPs and non-specific binding to the aptamer through electrostatic interaction. The sensing mechanism approach proposed for the detection of OTA is illustrated in Scheme 1. In the absence of OTA, the OTA aptamer is free and can combine with PDDA to form a “duplex” structure. AuNPs could not be aggregated and the mixture maintained a red wine color. However, in the presence of OTA, the state of OTA aptamers changed from a random coil structure to a “G-quadruplex” structure. Subsequently, PDDA induced the aggregation of AuNPs, leading to a change in the mixture color from wine red to blue.

### 3.2. Optimization of Experimental Conditions

**Figure 1:**
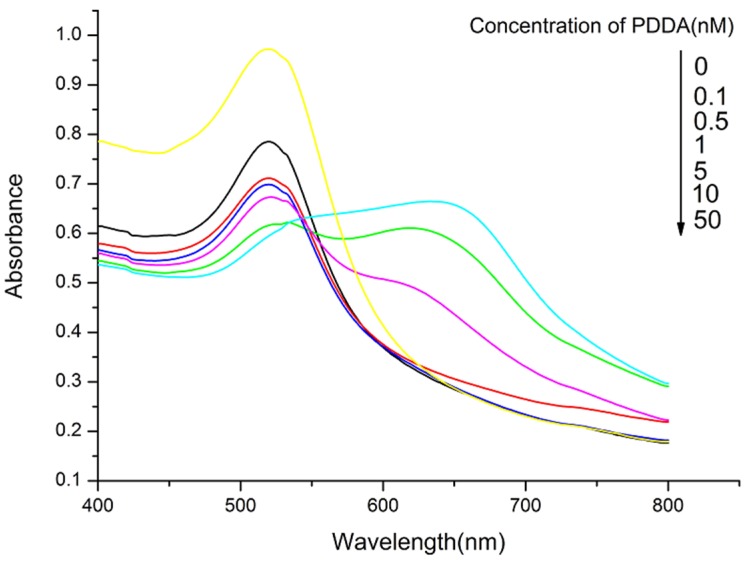
UV-vis absorbance spectra of AuNPs solutions in OTA binding buffer solution containing different concentrations of PDDA (0.1–50 nM).

**Figure 2:**
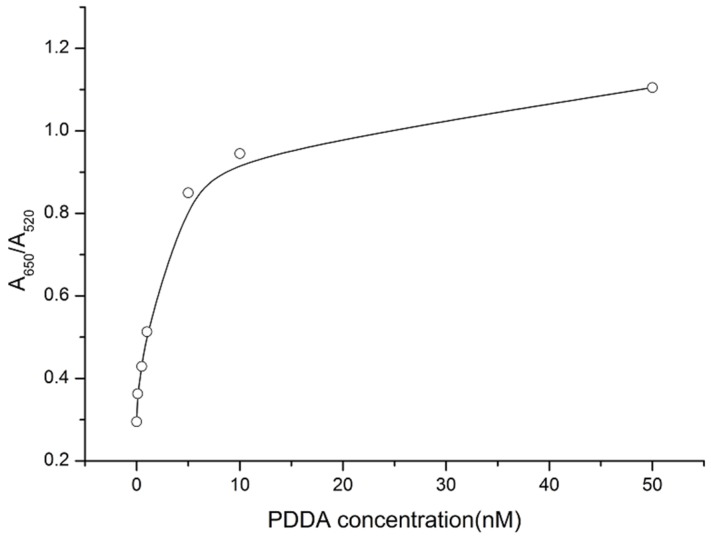
The variation in A650/A520 of AuNPs solutions treated with increasing concentrations of PDDA.

**Figure 3:**
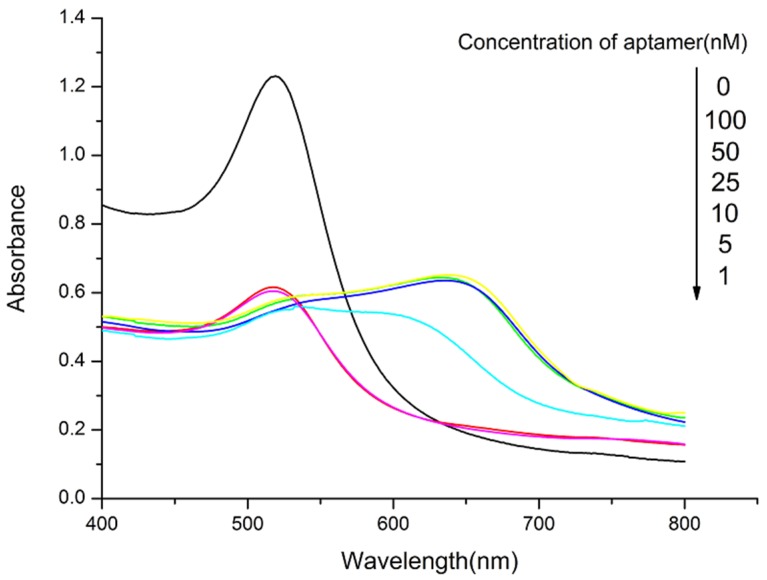
UV-vis absorbance spectra of AuNPs solutions in the presence of 5 nM PDDA treated with increasing concentrations of OTA aptamer.

To optimize the sensing conditions, varying concentrations of PDDA (0.1, 0.5, 1, 5, 10, and 50 nM) were added to AuNPs solutions of fixed concentration. The UV-vis absorbance values are shown in Figure 1. The UV intensity of AuNPs at 520 nm decreased with the addition of PDDA and the absorption peak was red-shifted to 650 nm. The relationships of the OTA concentration and the absorbance ratio (A650/A520) are shown in Figure 2. The results confirmed that 5 nM PDDA was suitable for aggregating all AuNPs. Thus, 5 nM PDDA was used in subsequent experiments. Various concentrations of the OTA aptamer (1, 5, 10, 25, 50, and 100 μM) were added to 1.5 mL plastic tubes containing 500 μL of 5 nM PDDA and 500 μL of AuNPs solution at a fixed concentration was added to each solution. As the aptamer concentration increased, the amount of PDDA bound to the aptamer also increased. AuNPs were aggregated by the remaining PDDA, causing the mixture to turn blue. As shown in Figure 3, 50 μM aptamer concentrations were suitable for the reaction and the subsequent detection of OTA.

### 3.3. Detection of Ochratoxin A with the Label-Free Aptamer-Based Assay

**Figure 4:**
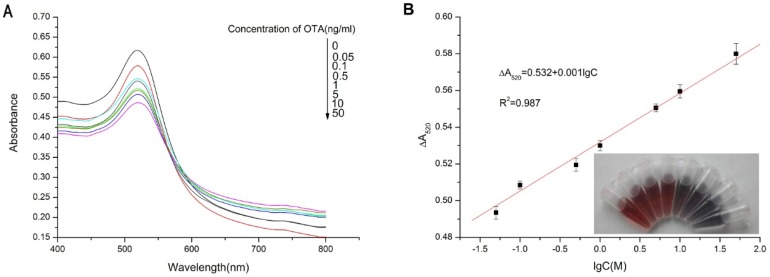
(**A**) Sensitivity of aptamer-based assay for OTA detection. The absorbance spectra of sensing solutions treated with 0, 0.05, 0.1, 0.5, 1, 5, 10 and 50 ng/mL OTA; (**B**) Calibration curve for the assay. Absorbance values were recorded at 520 nm as a function of the logarithm to base 10 of OTA concentration. The curve was fitted to a Hill plot with a correlation coefficient of 0.987. Visible colors of the reaction system with various concentrations of OTA (0, 0.05, 0.1, 0.5, 1, 5, 10, 50 ng/mL).

The optimized assay was applied for the detection of OTA in solutions of increasing OTA concentration from 0.05 to 50 ng/mL (Figure 4A). The increase of concentrations of OTA led to a decrease in the absorbance peak at 520 nm. As can be seen in Figure 4B, the ΔA520 (decrease in absorbance at 520 nm compared to the solution with the 0 ng/mL OTA) was proportional to the log value of the OTA concentration over the range of 0, 0.05, 0.1, 0.5, 1, 5, 10 and 50 ng/mL. The color of the reaction system changed from red to blue (Figure 4B). Figure 4B indicated that the ratio varied linearly with the concentration of OTA. Thus, the values of ΔA_520_ and the concentrations of OTA were fitted with the equation ΔA_520_ = 0.532 + 0.001 lgC, and the detection limit was estimated to be 0.009 ng/mL as calculated using the Standard Deviation and Slope approach. To compare with other methods, Table 1 summarizes the performance of the analytical methods for OTA determination.

**Figure 5:**
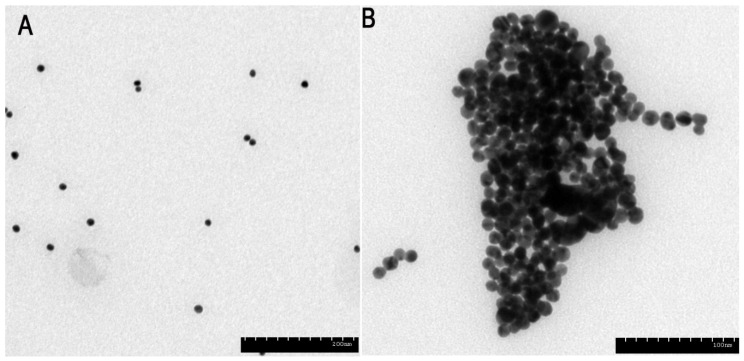
The variation in morphology of AuNPs through SEM. Images of AuNPs in solution containing PDDA and OTA-aptamer under the different concentrations of OTA of 0 ng/mL (**A**) and 1 ng/mL (**B**).

These results indicated that the optical property of the solution depends on the PDDA concentration, which is in turn conditioned directly by the amount of OTA, which makes it possible to detect OTA by a colorimetric assay. To confirm the supposed principle of such a strategy, SEM analyses were employed to characterize the aggregation of AuNPs. Figure 5 showed the morphology change of AuNPs through SEM. All these results were in good agreement with our assumption.

**Table 1:** Performance of analytical methods for Ochratoxin A (OTA) determination.

Method	Recognition Part	Limits of detection	Time	References
**TLC ^a^**	ND ^f^	0.05–0.93 ng·mL^−1^	>2 h	[6]
**HPLC-FLD ^b^**	ND ^f^	0.05–0.41 ng·mL^−1^	>2 h	[7,34]
**LC-MS/MS ^c^**	ND ^f^	0.01–0.18 ng·mL^−1^	>2 h	[35]
**ELISA ^d^**	Antibody	0.2–5.0 ng·mL^−1^	110 min	[10]
**FPIA ^e^**	Antibody	0.7 ng·mL^−1^	10 min	[36,37]
	Antibody	0.8 μg/kg	20 min	[36]
	Aptamer	2–5 ng·mL^−1^	45 min	[38]
**Aptasensor based on electrochemical assay**	Aptamer	0.02 pg·mL^−1^–0.07 ng·mL^−1^	30 min–1 h	[39,40]
**Aptasensor based on fluorescence assay**	Aptamer	3.6 ng·mL^−1^	30 min–1 h	[41]
**Aptamer-based assay based on AuNPs and poly diallyldimethylammonium chloride**	Aptamer	0.009 ng·mL^−1^	15 min	This work

^a^ TLC: Thin-layer chromatography; ^b^ HPLC-FLD: high-performance liquid chromatography: fluorescence detection; ^c^ LC-MS/MS: Liquid chromatography–mass spectrometry/mass spectrometry; ^d^ ELISA: Enzyme-Linked Immunosorbent Assay; ^e^ FPIA: Fluorescence polarization immunoassay; ^f^ ND: Not detected.

### 3.4. Detection Specificity

**Figure 6:**
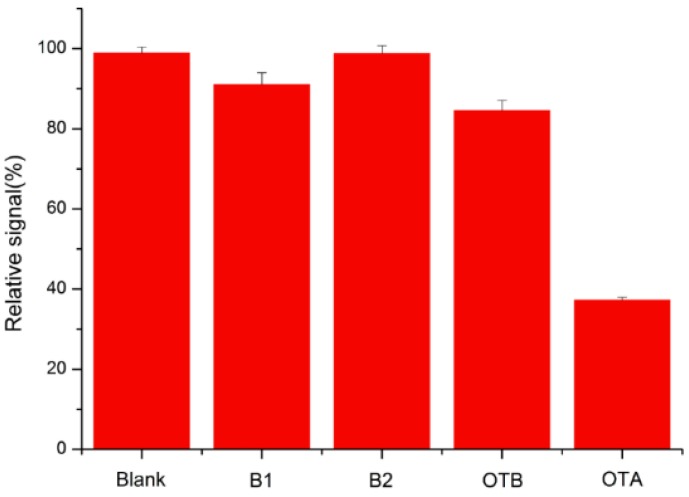
Selectivity of the aptamer-based assay for OTA detection. The concentrations of Aflatoxin B1, B2 and Ochratoxin B were both 0.5 ng/mL.

The selectivity of the method for OTA detection was also examined in order to evaluate the feasibility and reliability of the sensing system. Small-molecule toxins which could potentially compete with OTA were added at the same concentration as OTA to the sensing solution. The signals at 520 nm of Aflatoxin B1, B2 (AFB1, AFB2), Ochratoxin B (OTB) and OTA were calculated. As shown in Figure 6, the presence of AFB1 and AFB2 had a negligible effect on the detection and there was only about a 9.7% and 0.9% decrease of absorbance while the OTB resulted in about a 13.8% decrease compared to the blank sample. The OTB molecular structure represents as much a part of the OTA as it does the chlorine derivatives of OTA, which, to some extent, still possesses the binding ability with the OTA aptamer. Although the colors of the reaction systems were similar, OTA-induced aggregation was stronger than the interferences according to the specificity test. AFB1, AFB2 and OTB displayed a slight interference in the OTA detection.

### 3.5. Practicality of Ochratoxin A Detection in Liquor Samples

**Figure 7:**
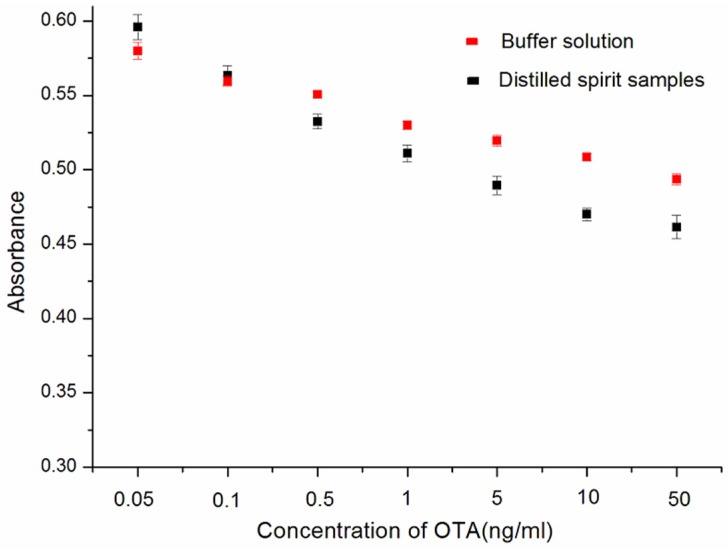
Determination of OTA spiked into distilled spirit samples.

In order to evaluate the potential applicability in practical samples, detection of OTA in the Chinese liquor sample was challenged by our aptamer-based assay. Different concentrations of standard solutions of OTA (0.05, 0.1, 0.5, 1, 5, 10, 50 ng/mL) were added into the 1% liquor. As shown in Figure 7, OTA in 100-fold diluted liquor was successfully detected with a wide linear concentration range from 0.05 to 50 ng/mL, and the absorbance was similar to that in the OTA buffer solution. These results indicate that the detection method can be applied to detect OTA in real samples with sufficient sensitivity.

## 4. Conclusions

In this work, a label-free aptamer-based assay for rapid detection of OTA was developed. PDDA was used to mediate AuNPs aggregation instead of sodium chloride, showing a higher sensitivity and preventing the interference of other cations which may be present in the solution of sodium chloride.

The analytical approach presents several advantages compared to current OTA detection methods. First, the reaction solution color changes from wine red to blue in the presence of OTA, which can be seen by the naked eye, so that test results can be acquired conveniently. Second, the limit of detection is as low as 0.009 ng/mL and the entire assay can be completed in less than 30 min, thereby achieving higher sensitivity and rapid screening of OTA with respect to other analytical methods for OTA determination (Table 1). Third, all the involved reagents are easy to prepare and reduce the cost of OTA detection compared with conventional analytical assays. As OTA has been detected in wine, liquor and beer, the aptamer-based assay was successfully applied to real samples of a Chinese liquor (Mao-tai) made from wheat and sorghum, without any pretreatment. Further research should be performed to show the applicability of this method for the detection of OTA in a large variety of foods.
